# Factors Controlling Changes in Epilithic Algal Biomass in the Mountain Streams of Subtropical Taiwan

**DOI:** 10.1371/journal.pone.0166604

**Published:** 2016-11-15

**Authors:** Yi-Ming Kuo, Hwa-Lung Yu, Wen-Hui Kuan, Mei-Hwa Kuo, Hsing-Juh Lin

**Affiliations:** 1 Laboratory of Basin Hydrology and Wetland Eco-restoration, School of Environmental Studies, China University of Geosciences, 430074 Wuhan, China; 2 Department of Bioenvironmental Systems Engineering, National Taiwan University, Taipei 10617, Taiwan; 3 Department of Safety, Health and Environmental Engineering, Ming Chi University of Technology, New Taipei City 24301, Taiwan; 4 Department of Entomology, National Chung Hsing University, Taichung 402, Taiwan; 5 Department of Life Sciences and Research Center for Global Change Biology, National Chung Hsing University, Taichung 402, Taiwan; 6 Biodiversity Research Center, Academia Sinica, Taipei 115, Taiwan; INRA, FRANCE

## Abstract

In upstream reaches, epilithic algae are one of the major primary producers and their biomass may alter the energy flow of food webs in stream ecosystems. However, the overgrowth of epilithic algae may deteriorate water quality. In this study, the effects of environmental variables on epilithic algal biomass were examined at 5 monitoring sites in mountain streams of the Wuling basin of subtropical Taiwan over a 5-year period (2006–2011) by using a generalized additive model (GAM). Epilithic algal biomass and some variables observed at pristine sites obviously differed from those at the channelized stream with intensive agricultural activity. The results of the optimal GAM showed that water temperature, turbidity, current velocity, dissolved oxygen (DO), pH, and ammonium–N (NH_4_–N) were the main factors explaining seasonal variations of epilithic algal biomass in the streams. The change points of smoothing curves for velocity, DO, NH_4_–N, pH, turbidity, and water temperature were approximately 0.40 m s^-1^, 8.0 mg L^-1^, 0.01 mg L^-1^, 8.5, 0.60 NTU, and 15°C, respectively. When aforementioned variables were greater than relevant change points, epilithic algal biomass was increased with pH and water temperature, and decreased with water velocity, DO, turbidity, and NH_4_–N. These change points may serve as a framework for managing the growth of epilithic algae. Understanding the relationship between environmental variables and epilithic algal biomass can provide a useful approach for maintaining the functioning in stream ecosystems.

## Introduction

The upstream reaches of the Dajia River, located in the Wuling basin of the Shei-Pa National Park in central Taiwan at approximately 1800 m above sea level, are the only habitats of the Taiwanese masu salmon (Formosan landlocked salmon; *Oncorhynchus masou formosanus*). Because of its limited population and narrow distribution, the The International Union for Conservation of Nature (IUCN) listed the Taiwanese masu salmon as a critically endangered species in 1996 [1]. Epilithic algae are one of the major primary producers in streams and play major roles in controlling energy flow of food webs in stream ecosystems [2–4]. However, the overgrowth of epilithic algae may deteriorate water quality [5]. The variation of epilithic algal biomass may indirectly affect the distribution and population of the Taiwanese masu salmon.

The production and dynamics of an epilithic algal composition in stream ecosystems are also largely influenced by physical variables such as geochemical conditions, flow rate, current velocity, light, and water temperature [6–8]. Water quality variables (electrical conductivity, pH, total dissolved solids, chemical oxygen demand, biochemical oxygen demand, and nutrients such as phosphorus and nitrogen from the surrounding lands) also play major roles in regulating the production rate and species composition of epilithic algae in streams [9–13]. However, in complex stream ecosystems, the dynamics of epilithic algal biomass may nonlinearly interact with the combination of abiotic and biotic factors. A technique should be employed to describe the nonlinear relationships between epilithic algal biomass and aforementioned factors.

A generalized additive model [14], which is an extension of a generalized linear model [15], enables analyzing nonlinear effects such as additive functions and smooth components in explanatory variables. GAM has been extensively applied in ecological studies such as modeling habitat suitability and ecological relationship [16–19], algal bloom analysis in Lake Taihu [20], landslide susceptibility analysis [21], geomorphological distribution modeling in a complex terrain [22–23], and air pollution research [24–25]. Ecological data tend to extremely noisy and heterogeneous. Therefore, the GAM may facilitate improving our understanding of dynamics and controlling factors of epilithic algal biomass.

Despite the availability of strong evidence of effects of nutrients and environmental factors on epilithic algal growth in numerous rivers, most studies on epilithic algae have been conducted in low-altitude streams of temperate climate regions [26–29]. In the current study, chlorophyll a (Chl-a) was used to estimate epilithic algal biomass [30]. Water quality, environmental variables, and Chl-a were examined in high-altitude streams of subtropical climates. The specific study objectives were to apply the GAM to determine which key abiotic and biotic factors (as explanatory variables) considerably influence epilithic algal biomass and provide insight into how algal biomass nonlinearly responds to the data range of aforementioned key factors. Evaluating and maintaining the growth of epilithic algae, which consequently provides a habitat with sufficient food for Taiwanese masu salmon, necessitates the long-term monitoring of the temporal variations in epilithic algal biomass and key controlling factors.

## Materials and Methods

### Study area

**Fig 1:**
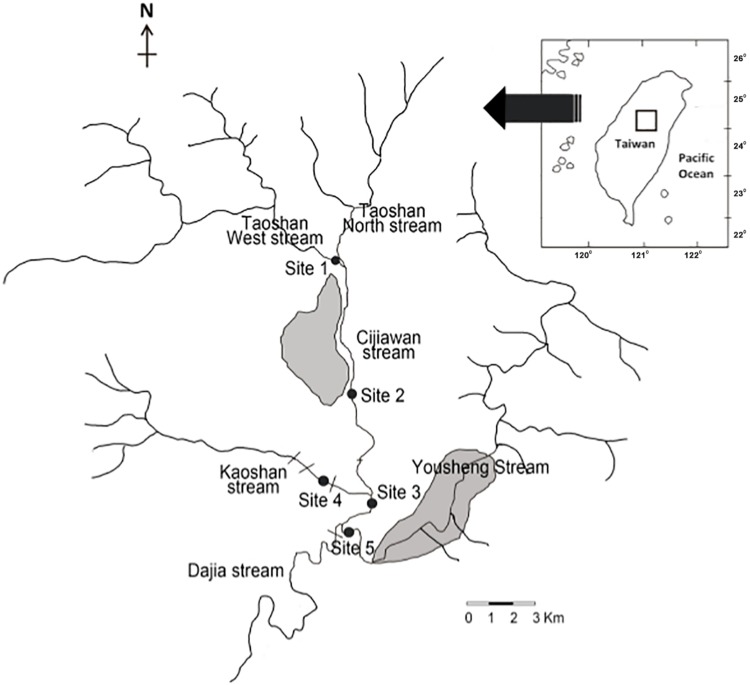
Monitoring sites in the upstream reaches of the Dajia River in the Shei-Pa National Park in Wuling, Taiwan. Agricultural areas were shown in dark grey.

The upstream reaches of the Dajia River located in the Wuling basin of the Shei-Pa National Park comprises 3 third-order streams [Chichiawan (CCW), Yousheng (YS), and Kaoshan (KS) Streams] and 2 second-order streams [Taoshan West (TW) and Taoshan North (TN) Streams] (Fig 1). The CCW and KS Streams are the only habitats of the Taiwanese masu salmon. The CCW, YS, and KS Streams are characterized as short, straight, and steep channels, respectively, and are often influenced by heavy storms. The mean discharge of the upstream Dajia River in the dry season was 1.84–2.30 m^3^·s^−1^ and that in the wet season was 2.58–2.96 m^3^·s^−1^ [31]. The mean annual water temperature was 12°C, ranging from 10°C in winter to 18°C in summer [32]. The mean annual precipitation is 1640 mm and the mean monthly rainfall typically did not exceed 40 mm in the dry season of October–April. However, the mean rainfall frequently exceeded 300 mm·month^−1^ in the wet season of May–September [5].

Epilithic algae were monitored at 5 sites (Fig 1) that reflected the different cover levels of riparian vegetation and agricultural activities (Table 1). The permission for setting up these 5 monitoring sites was issued by the Shei-Pa National Park Authority. Diatoms were the most dominant taxa and contributed 85% of the total cell numbers of the epilithic algal communities in the streams of the Wuling basin [32]. Site 1 was located in the TW Stream. The watershed of the TW Stream vegetated by a pristine riparian forest. Sites 2 and 3 were located in the central and down reaches of the CCW Stream, respectively. Yeh [33] reported that the streambed comprised a high proportion of pebble (42%) in winter, but was dominated by rubble (26%) and boulders (21%) in summer. The upper reach of the CCW Stream is bordered by a riparian forest, but the central reach, where Site 2 was located, is developed for agriculture including an area of 104 ha of vegetables, apples, peaches, and pears [32]. Site 4 was located in the KS Stream in the pristine down reach of the CCW Stream. According to Yeh [33], the streambed was dominated by pebble (39%) and rubble (27%) in winter, but had a high proportion of boulders (44%) in summer. The surroundings of the KS Stream are vegetated by natural forests (no agriculture); therefore, this stream can be considered to be in a pristine state [34]. Site 5 was located in the downstream reach of the YS Stream. The streambed is dominated by gravel (39%) and pebble (39%) [33]. The YS Stream demonstrates relatively high nutrient concentrations and has been channelized and developed for agriculture since 1970s. Since Site 2 bordered by a riparian forest, Sites 1–4 were considered pristine forests, whereas Site 5 can be attributed to the agricultural activity.

**Table 1:** Comparison of site characteristics in the upstream watershed of the Dajia River. Locations of study sites are shown in Fig 1. Sites 2 and 3 are located in CCW Stream.

	Site 1	Site 2	Site 3	Site 4	Site 5
Elevation (m)	1900	1790	1742	1776	1770
Channel slope (m km^-1^)	41.6	128	132	140	68
Channel width (m)	3–4	30–35	23–30	<10	10–15
Channel condition	Natural	Natural	Natural	Natural	Channelized
Surrounding land use(refer to Tsai et al., 2013)	Pristine forest	Moderate agricultural activity, some natural riparian forest	Pristine forest	Pristine forest	Intensive agricultural activity, no natural riparian vegetation
Stream attribute (length/area)	TW Stream (13.8 km/41.6 km^2^)	CCW Stream (15.3 km/76 km^2^)		KS Stream (10.6 km/40 km^2^)	YS Stream (11.4 km/31 km^2^)

### Sample collection

At each site, samples of epilithic algae were collected bimonthly from randomly selected cobbles (*n* = 6) in the riffle zone during June 2006–June 2011 (5 y). On each cobble, a 12.5-cm^2^ transparent steel frame was placed to define a sampling area of an algal patch. Four algal patches (total surface area of 50 cm^2^ on each cobble) were scraped with a toothbrush, and the toothbrush and cobbles were subsequently washed with 50–100 mL of filtered stream water [32]. In the laboratory, the algal samples were homogenized and then centrifuged at 3500 rpm for 10 min to concentrate them to 5 mL. A 3-mL subsample was extracted for Chl-a concentration in 90% acetone for 24 h at 4°C in the dark and analyzed spectrophotometrically [35].

At each sampling site, the water temperature (TEMP), pH, conductivity (EC), turbidity, and dissolved oxygen (DO) of the stream were measured in situ by using YSI 600XLM multiparameter monitoring systems and portable meters (YSI Inc., Yellow Springs, OH, USA). The current velocity was measured at the upstream 1 cm of the selected cobble using a velocimeter (FlowTracker handheld-ADV, SonTek/YSI Inc., San Diego, CA, USA). Water samples were immediately placed on ice in a cooler and returned to the laboratory for analyzing nitrate–nitrogen (NO_3_–N), ammonium–nitrogen (NH_4_–N), sulfate (SO_4_^2−^), orthophosphate (PO_4_^3−^), total organic carbon (TOC), and dissolved silicate (SiO_2_) according to the standard methods of the American Public Health Association [36]. Aquatic insects were collected from 6 random samples using a Surber net sampler (30.5 × 30.5 cm net with a mesh size of 250 μm) at each site. Insects were preserved in 75% ethyl alcohol and then determined the biomass and identified organisms to the lowest possible taxonomic level using available keys [37] in laboratory. No endangered or protected species were involved in this study.

### Generalized additive model

The GAM is a regression model that assumes that response variables are dependent on the smoothing splines of independent variables instead of linear coefficients [14]. The GAM used in this study is as follows:

$$g\left(\mu_{i}\right)=\alpha +\sum_{j=1}^{n}S_{j}\left(X_{ij}\right)+factor\left(ENV_{l}\right)+\sum_{j=1}^{n}S_{j}\left(X_{ij}\right):factor\left(ENV_{l}\right)$$ (1)

where *g* is the specified link function. In addition, *μ*_*i*_ = E(*Y*_*i*_) is the expected values of the response variable (*Y*_*i*_), where *Y*_*i*_ is the *i*th Chl-*a* concentration; α is the intercept; and *X*_*ij*_ is the *i*th value of explanatory variable *X*_*j*_. The *i* ranges from 1 to 135 because each site had data of 27 samples. *S*_*j*_(*X*_*ij*_) is the smooth function (smoothing spline) of *i*th value of explanatory variable (*X*_*j*_), and *n* is the total number of explanatory variable. Moreover, *ENV*_*l*_ is a nominal explanatory variable representing types of surrounding environments. The term *factor*(*ENV*_*l*_) adds or subtracts only a constant value from the smoother.

### GAM variables

The Chl-*a* concentration at the 5 sites served as the response variable in the GAM. Water quality variables (SO_4_^2−^, PO_4_^3−^, NH_4_–N, NO_3_–N, TOC, SiO_2_, pH, EC, turbidity, and DO) and environmental variables (canopy cover, water temperature, current velocity, and the number of aquatic insects) served as explanatory variables in the GAM. Although the Site 2 has moderate agricultural activity, the natural riparian vegetation along the river band can retard nonpoint source pollution to degrade the water quality. Sites 1–4 can be attributed to the Environment 1 (natural riparian vegetation) and Site 5 can be attributed the Environment 2 (anthropic disturbance without riparian vegetation). The Environments 1 and 2 served as nominal explanatory variables are used in GAM models for investigating the effects of surrounding environment conditions on stream Chl-*a* concentration.

### Analytical procedure

A crucial step in applying GAMs is selecting an appropriate level of the “smoother” for a predictor. This can be achieved by specifying the level of smoothing by using the concept of effective degrees of freedom. A reasonable balance must be maintained between the total number of observations and the total number of degrees of freedom used when fitting the model. During model selection in the GAM, the Akaike information criterion [38] was used to determine the optimal GAM model. The types of the specified link function and distributions of response variable were considered. In each backward selection step, cross-validation was applied to estimate the optimal degrees of freedom for each smoother. Finally, variance inflation factor (VIF) analysis [39] was performed to detect multicollinearity in each set of explanatory variables. In the optimal GAM model, the VIF values of explanatory variables were controlled within 5 [40]. Using multicollinear explanatory variables would generate an error or warning message in GAM outputs. GAM was employed using the Brodgar Version 2.7.4 statistical package (Highland Statistics Ltd., Newburgh, UK), which is based on the statistical software language “R” Version 3.0.2. The R library “mgcv” allows for the automatic application of the cross-validation method in the GAM.

## Results

### Descriptive statistical analysis

**Fig 2:**
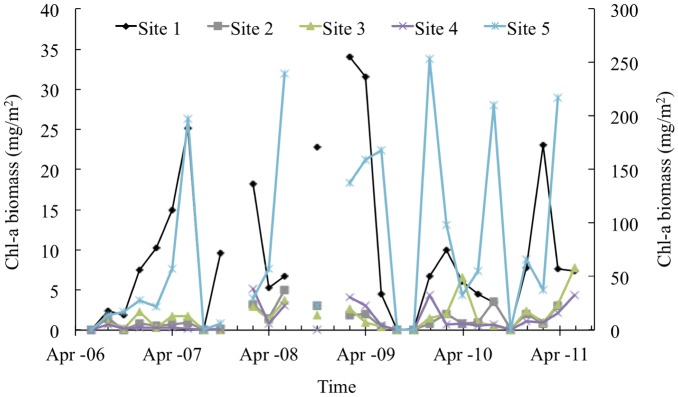
Seasonal variations in the Chl-*a* concentration for bimonthly observations at the 5 sites during 2006–2011. Y-axis in the right is only for Site 5.

**Fig 3:**
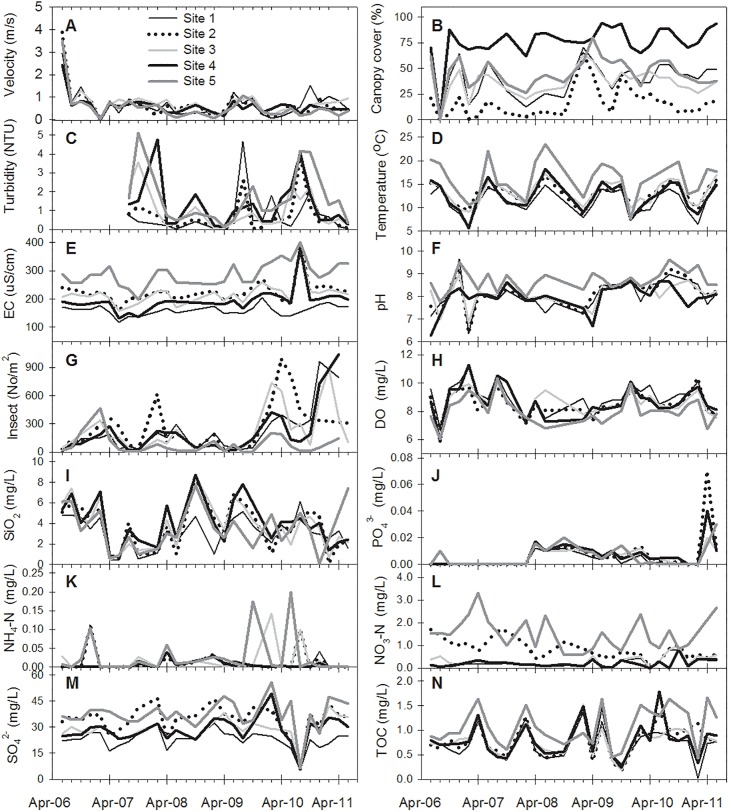
Seasonal variations in abiotic and biotic variables for bimonthly observations at the 5 sites during 2006–2011.

Table 2 showed the mean and coefficient of variation (CV) of the response and explanatory variables for each site. High CVs (CV > 50%) were observed for the concentrations of Chl-*a*, SiO_2_, PO_4_^3−^, NH_4_–N, and NO_3_–N, velocity, turbidity, and number of aquatic insects. The highest Chl-*a* concentration determined in the YS Stream (Site 5) surrounded by intensive agricultural activities was 6–9 times higher than that in other streams (Fig 2). Fig 3 illustrates seasonal fluctuations in explanatory variables during the study period and the magnitudes of the water temperature, EC, pH, NO_3_–N, and SO_4_^2−^ at Site 5 were also higher than other four sites. Canopy cover was highest at Site 4, surrounded by a pristine forest. Fluctuations in TOC, SO_4_^2−^, and SiO_2_ followed the same patterns at all sites before April 2009, after which they considerably varied until the end of the study period.

**Table 2:** Mean and coefficient of variation (CV) of variables for each site. Units of the last 7 variables are mg L^-1^.

Variables	Site 1		Site 2		Site 3		Site 4		Site 5	
	Mean	CV	Mean	CV	Mean	CV	Mean	CV	Mean	CV
Chl-a (mg m^-2^)	9.70	99.0	8.43	132.2	13.14	108.1	9.30	127.7	78.80	107.3
Velocity (m s^-1^)	0.70	83.8	0.606	69.6	0.77	79.6	0.60	68.7	0.57	112.4
Canopy cover(%)	42.2	15.4	75.39	23.5	38.5	11.7	79.0	9.38	47.0	12.0
Turbidity (NTU)	0.72	144.8	1.693	121.6	1.24	140.1	1.63	125.0	5.63	189.1
Temperature (°C)	11.42	23.7	12.36	24.5	13.33	18.9	12.45	24.2	16.16	23.8
EC (μS cm^-1^)	176.1	46.3	194.5	22.5	218.2	18.7	194.6	22.1	289.1	14.7
pH	8.03	9.4	7.96	7.40	8.12	6.5	7.97	7.3	8.70	5.3
Aquatic insect (No. m^-2^)	214.5	110.4	199.7	120.8	221.6	107.5	199.7	120.8	81.67	130.8
DO	8.95	11.5	8.72	12.97	8.60	10.1	8.70	12.8	8.26	16.6
SiO_2_	3.116	50.1	4.419	63.4	3.734	58.2	4.345	63.9	3.766	78.3
PO_4_^3-^	0.004	150.0	0.075	140.3	0.042	158.8	0.072	176.6	0.004	148.0
NH_4_-N	0.011	170.3	0.011	166.3	0.015	176.5	0.009	181.1	0.019	180.5
NO_3_-N	0.225	92.0	0.200	86.5	0.317	73.2	0.206	83.8	1.475	46.7
SO_4_^2-^	22.70	23.2	28.33	26.1	28.93	22.8	28.40	25.6	37.26	24.1
TOC	0.710	43.3	0.812	46.7	0.778	39.3	0.815	45.7	1.082	37.0

### GAM results

Several GAM models combining with various explanatory variables were executed; however, only the numerical outputs of significant explanatory variables in the optimal model were listed in Table 3. The optimal GAM involved a Poisson distribution and log link function. The optimal model explained 91.0% of deviance, indicating that 91.0% of the total sum of squares was explained by the optimal GAM (R^2^ = 0.942 and RMSE = 5.12). The residual variance was 309 and AIC was 607.

**Table 3:** The numerical outputs (parametric coefficients and approximate significance of smooth terms) of the optimal GAM model.

Nominal variables	Parametric coefficients			
	Estimate	Standard errors	*t*-value	Pr(>|*t*|)^#^
Intercept	2.234	0.054	41.28	<2e-16 ***
factor(*ENV*_*2*_)	1.705	0.155	10.97	<2e-16 ***
Smoothers	Approximate significance of smooth terms			
	edf^$^	Ref.df^$^	Chi Square	*p*-value^#^
s(Velocity)	2.656	3.181	39.54	2.37e-08 *** ******
s(pH)	4.000	4.000	94.35	< 2e-16 ***
s(DO)	2.192	2.815	55.73	8.56e-12 ***
s(Temperature)	1.998	2.000	59.93	9.70e-14 ***
s(NH4)	2.806	2.961	20.05	1.02e-05 ***
s(Turbidity):factor(*ENV*_*1*_)	2.992	3.000	56.78	6.51e-11 ***
s(Turbidity):factor(*ENV*_*2*_)	3.000	3.000	84.99	< 2e-16 ***

^$^edf: estimated degree of freedom; Ref.df: estimated degree of freedom for reference

^#^Significant code: ***: 0.001.

The optimal GAM model showed that the water temperature, turbidity, current velocity, DO, pH, and NH_4_–N were the main factors explaining the long-term dynamics of Chl-*a* concentration in the study area (Table 2). Among six significant explanatory variables, DO is related to the photosynthetic production and respiratory consumption of the epilithic algal biomass. DO is the result from epilithic algal growth rather than the factor affects their variations. However, other five variables can be attributed to water quality and environmental variables. The VIFs for these six explanatory variables (1.05≤ VIF ≤ 1.64) did not exceed the VIF threshold. Table 3 shows the smoothers for each significant variable and all smoothers are highly significant at the 0.001 level. Since the turbidity has a high CV, an individual smoother was used to describe the relationships between the turbidity and epilithic algal biomass for each Environment. The estimated degrees of freedom (edf shown on the Y-axis) for all smoothers are in the ranges of 2.00 and 4.00, indicating that these variables are moderately nonlinear relationships with Chl-*a* concentration (Table 3).

The intercept had a value of 2.234 (Table 2) and was significantly different from 0 at the 0.1% level. The notation “factor (Environment 2)” denotes the Chl-*a* concentration at Environment 2. Its estimated regression parametric coefficient was 1.705, indicating that the Chl-*a* concentration at Environment 2 was 1.705 mg·m^−2^, higher than that at Environment 1. The *p-*values for the environment levels indicated which monitoring environments were significantly different from the baseline, namely Environment 1. The Chl-*a* concentration observed at Site 5 (Environment 2) with intensive agricultural activity was significantly different from those observed at the other four sites (Environment 1 with natural riparian vegetation/forest) at the 0.1% level.

## Discussion

### Relationships between epilithic Chl-a concentration and explanatory variables

The mean velocities at the 5 sites ranged from 0.55 to 0.70 m·s^−1^, which can be considered moderate to high velocity [41–42]. The Chl-a concentration was not affected by velocity when velocity is smaller than 0.55 m·s^−1^. However, an increasing velocity reduces epilithic algal biomass once the velocity is greater than the threshold of 0.55 m·s^−1^. Water velocity was the important variable regulating epilithic algal composition [43]. Tsai et al. [44] developed a process-based model to investigate how storm-induced velocity influenced the variations of epilithic algal biomass in the CCW Stream. They determined that the algal biomass was considerably reduced by a high storm-induced velocity. A high velocity may cause rocks to tumble and sediment substrata on the streambed to be scoured [45–46] and change the community structure of epilithic algae [47]. However, a low or moderate velocity may reduce the thickness of the diffusion boundary layer and increase nutrient uptake [41–42], and consequently increase algal biomass or biodiversity [48]. Heath et al. [49] showed that decreased velocity reduced nutrient dilution capacity and then indirectly increased benthic cyanobacterial blooms. Epilithic algal biomass was increased with velocity (velocity ≤ 0.60 m·s^−1^), but further increase in velocity reduced the biomass [50]. Therefore, variations in velocity result in different relationships with epilithic algal biomass because once the flow velocity reaches a critical level, the shear stresses induce the algae to detach from the epilithic algal mat [51].

CV values of turbidity at these 5 sites are greater than 120 showing high fluctuations. Therefore, turbidity may have a complicated relationship with epilithic algal biomass (Table 2). For both Environments 1 and 2, epilithic algal biomass was increased with turbidity (when turbidity <0.60 NTU) and then decreased with turbidity after that value. However, at Environment 1 only four observations (shown as ticks on the X axis) of epilithic algal biomass were increased with turbidity (when turbidity >2.3 NTU). Increases in turbidity can reduce the available light reaching the streambed and thus diminish the stream temperature, possibly affecting epilithic algal growth rates [52–53]. Therefore, turbidity considerably influenced variations in net community production and respiration rates of epilithic algae [54]. However, Figueroa-Nieves et al. [55] reported that the interaction between hydrology and turbidity might control algal biomass in nutrient-rich agricultural streams. The effects of nutrients and light availability on epilithic algal biomass are evident only after accounting for the large-scale constraints of land use, such as the levels of riparian vegetation and agricultural activity [41].

**Fig 4:**
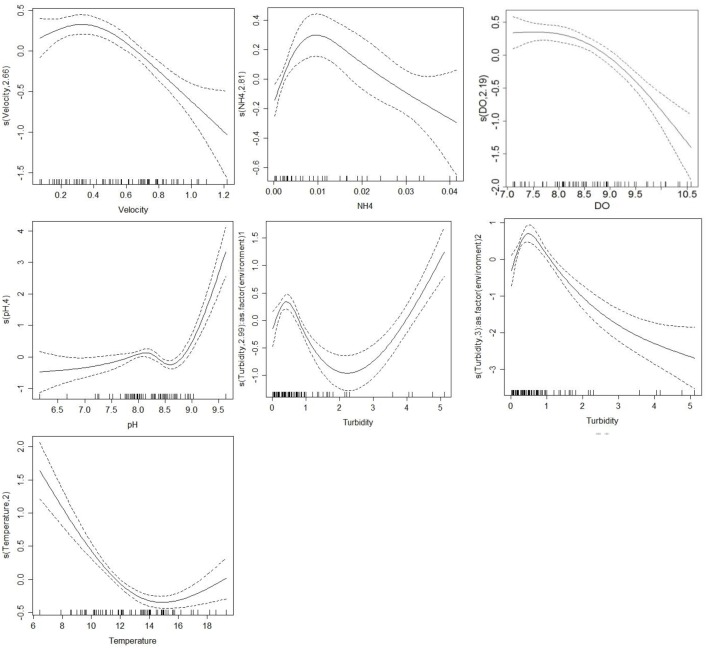
Smooth function for each critical environmental factor on Chl-*a* concentration. Numbers in brackets in the captions of the Y-axis are the edf of the smooth curves. Solid lines represent smoothers and dotted lines represent 95% confidence intervals.

Water temperature sharply and negatively (when water temperature <14°C) correlated with epilithic algal biomass (Fig 4). However, when water temperature was greater than 15°C, the curve became a slightly increasing trend with an uncertainty expanded (i.e. the confidence interval is becoming wider). Two previous studies conducted in the streams of the Wuling basin support our results. Yu and Li [32] indicated that abundances of some epilithic algal species (diatoms such as *A*. *atomus* and *Planothidium*) in the streams were negatively correlated with water temperature. Tsai et al. [41] found that increasing temperature stimulated algal growth at sites with a moderate or low canopy cover, but it restrained algal growth at sites with a dense canopy cover. The epilithic diatoms are dominated at lower temperatures and consequently are the most abundant group during the winter and spring [56]. Variations in water temperature and canopy cover probably cause different correlations between temperature and algal growth rates.

In this study, the DO concentrations during the study period were greater than 6.5 mg/L, which can be attributed to “good” water quality [57]. Fig 4 shows that the epilithic Chl-a concentration is not related to DO when the DO level ranges between 7.0 and 8.0 mg/L and is decreased with DO when the DO level is higher than 8.0 mg/L. DO levels in surface waters are affected by a number of interacting processes including: photosynthesis and respiration of aquatic organisms [58], bacterial respiration, carbonaceous and nitrogenous deoxygenations, nitrification, reaeration, and sediment oxygen demand. Epilithic algae play an important role in photosynthetic production and respiratory consumption of DO [59], and consequently regulate the diurnal variation in DO concentration [60]. In addition, higher TOC and lower DO levels were obviously found at Site 5 indicating that bacteria consumed oxygen while breaking down organic matter, and then reduced DO levels in the Wuling streams.

Fig 4 shows that the epilithic Chl-*a* concentration is negatively and linearly correlated with NH_4_–N when NH_4_–N is greater than 0.010 mg/L (edf is 2.987). However, in most samples (82%) the NH_4_–N concentrations were less than 0.010 mg/L and positively related to epilithic Chl-*a* concentration. The NH_4_–N concentrations in the YS Stream (Site 5) derived mainly from agricultural runoff were mostly higher than other four sites. Ammonium is readily bioavailable for algae uptake. Therefore, ammonium significantly affects epilithic Chl-*a* concentration in this study. Tien et al. [61] determined that epilithic algae in biofilms were negatively correlated with ammonium concentrations and positively correlated with pH in the Erh-Jen River, which showed the similar correlations in this study.

The recharge of agricultural nonpoint source pollution associated with lime-rich fertilizers can result in increased pH in aquatic systems. The pH affects most chemical and biological processes in water, and is one of the most important environmental factors limiting the distribution of species in aquatic habitats. Average pH values at the study sites were slightly alkaline, ranging from 7.96 to 8.70. Fig 4 showed that epilithic algal biomass sharply increases with the pH when the pH value is greater than 8.8; otherwise, the algal biomass slightly increases with the pH when pH value is less than 8.8. The change in pH affects the aqueous equilibria (such as ammonia, hydrogen sulfide, and dissolved metals) and is directly related to the availability and absorption of nutrients [62]. As algal bloom, more carbon dioxide is removed by photosynthesis of epilithic algae than is added by respiration, thus elevating pH levels in water and leading to the enhance of the algal growth [63]. Therefore, Kivrak and Uygun [13] also found that some of diatom taxa were positively correlated with pH. Epilithic algal species composition was significantly correlated with pH in a pristine subalpine stream [64]. pH is a major environmental variable determining diatom distributions in water bodies in temperate areas [65] and exerts a strongest effect on epilithic algal species composition [66].

### Suitable habitat for epilithic algae

Site 5 located in an area with intensive agricultural activity has the highest Chl-a concentration. The contribution of nutrient enrichment on epilithic algal growth under certain conditions is limited by environmental variables such as temperature and light availability. Complex habitats with diverse combinations and ranges of water quality and environmental variables result in different correlations between these variables and epilithic algal biomass.

The change points of smoothing curves for velocity, DO, NH_4_–N, pH, turbidity, and water temperature were approximately 0.40 m s^-1^, 8.0 mg L^-1^, 0.01 mg L^-1^, 8.5, 0.60 NTU, and 15°C, respectively. When aforementioned variables were greater than relevant change points, the epilithic algal biomass was sharply increased with pH and water temperature, and sharply decreased with water velocity, DO, turbidity, and NH_4_–N. However, epilithic algal biomass at the Environment 1 (Sites 1–4) was increased with turbidity when turbidity was greater than 2.30 NTU. These change points may serve as a framework for managing the growth of epilithic algae, especially diatoms, in the Wuling streams. Many streams suffer from overgrowth of epilithic algae due to anthropogenic nutrient loading [67]. If administrators of the Shei-Pa National Park understand the relationship between environmental variables and epilithic algal biomass, the optimal levels of epilithic algal biomass in the streams can be effectively maintained.

## Supporting Information

S1 File This file contains the raw data used in modeling. (XLSX)
